# Women’s perception of pre-hospital labour duration and obstetrical outcomes; a prospective cohort study

**DOI:** 10.1186/1471-2393-14-182

**Published:** 2014-05-30

**Authors:** Patricia A Janssen, Sandra Weissinger

**Affiliations:** 1 University of British Columbia, School of Population and Public Health, Child and Family Research Institute, 2206 East Mall, Vancouver, BC V6T-1Z3, Canada; 2 University of British Columbia, Faculty of Medicine, Midwifery Program, Vancouver, Canada

**Keywords:** Early labour, Cesarean, Obstetrical outcomes, Newborn outcomes

## Abstract

**Background:**

Progress during early labour may impact subsequent labour trajectories. Women admitted to hospital in latent phase (<3 cm cervical dilation) labour have been shown to be at higher risk of obstetrical interventions.

**Methods:**

We conducted a secondary analysis of data from a randomized controlled trial of 1247 healthy nulliparous women in spontaneous labour at term with a singleton fetus in cephalic presentation at seven hospitals in Southwestern British Columbia. We computed relative risks and their 95% confidence intervals to examine our primary outcome of cesarean section and secondary outcomes including obstetrical interventions and maternal and newborn outcomes according to women’s perception of length of pre-hospital labour. Women were asked on admission to hospital how long they had been experiencing contractions prior to coming to hospital.

**Results:**

Women indicating that they had been in labour for 24 hours or longer at the time of hospital admission were at elevated risk for cesarean birth, relative risk (RR) 1.40, (95% Confidence Intervals 1.15-1.72), admission with a cervical dilation of 3 cm or less, RR 1.21 (1.07-1.36), more obstetrical interventions including continuous electronic fetal monitoring RR 1.11 (1.03-1.20), augmentation of labour RR 1.33 (1.23-1.44), use of narcotic RR 1.21 (1.06-1.37) and epidural analgesia RR 1.18 (1.09-1.28). Adverse neonatal outcomes did not differ apart from a significant increase in meconium-stained amniotic fluid RR 1.60 (1.09-2.35).

**Conclusions:**

A single question asked of women on presentation to hospital was an important predictor of cesarean birth and may have utility in identifying women who would benefit from close observation and more active management of labour.

## Background

In Canada and the United States, cesarean section rates have risen to 26.9% [1] and 32.8% [2] in 2010. While this rise has been attributed to the compounding effect of repeat cesarean births and an increase in obesity [3], hypertension [4], and multiple births [5], the factors precipitating cesarean birth among apparently healthy women remain relatively unexplored. The leading indication for primary cesarean is dystocia [6]. Research to date focused on low-risk nulliparous women suggests that events during early labour impact subsequent labour trajectories including the diagnosis of dystocia [7]. Increased pain or distress in early labour is associated with slow progress and need for analgesia in active labour [8]. Women admitted to hospital in latent phase (<3 cm cervical dilation) have been shown to be at higher risk of obstetrical interventions, including electronic fetal monitoring, epidural analgesia, oxytocin, and caesarean section, than those who are admitted in active labour [9-13]. Approaches to early labour care including home visits [14,15], standardized definitions of labour onset [16] or targeted interventions to manage discomfort [17] have been evaluated in randomized controlled trials and have not been shown to influence labour outcomes.

The Early Labour Assessment and Support at Home Trial (ELASH) collected detailed data about women’s labour experience prior to hospital admission [14]. Women presenting to hospital were asked by nurses how long they had been in labour and their responses were documented without further prompting or questioning. In the current study we tested the hypothesis that women’s perceptions that their labour had been underway for more than 24 hours at the time of hospital admission was associated with cesarean section and other obstetrical outcomes. Additionally, we compared neonatal outcomes among women with ≥ 24 versus < 24 perceived hours of labour prior to hospital admission.

## Methods

**Figure 1:**
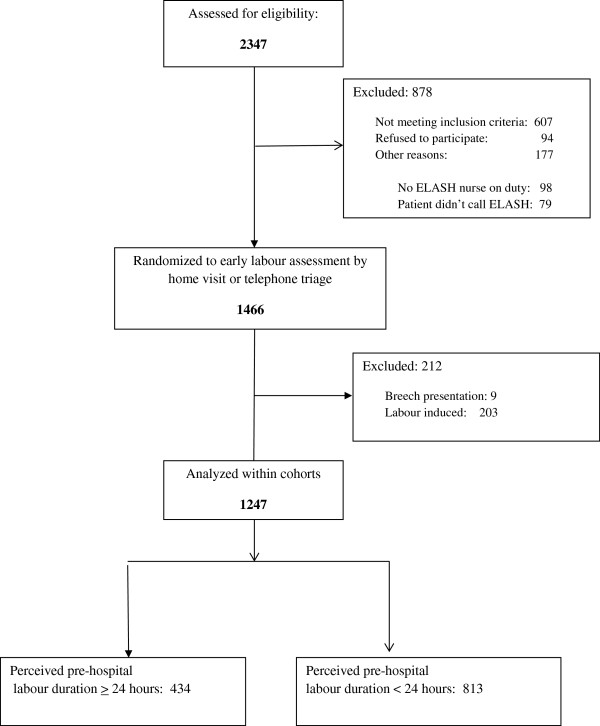
Flow diagram for recruitment.

We undertook a secondary analysis of data collected for the ELASH trial of assessment and support in early labour by obstetrical nurses via telephone versus home visits. Methods for this study have been reported elsewhere in detail [14], but in brief this study recruited healthy nulliparous women, between the ages of 16 and 42, at 37–41 completed weeks of gestation, carrying a singleton fetus in vertex position without pre-existing medical conditions or any conditions arising in pregnancy that precluded their physicians from advising them to remain at home in early labour. In the current study we excluded 212 women who were being induced on an out-patient basis with cervical prostaglandins or were found to have a baby in the breech position during labour from the original 1459 for a final sample of 1247 (Figure 1). Thus, our sample met the criteria of the Robson Classification, Category 1 [18].

Women were asked by the admitting nurse how many hours they had been experiencing contractions prior to their final hospital admission. Their answers were recorded verbatim without question. We chose to categorize our exposure into ≥ or < 24 hours of pre-hospital labour as we believe that most clinicians would choose to admit women for labour augmentation after 24 hours. A recently published consensus statement from the American College of Obstetricians and Gynecologists defines prolonged latent phase labour as lasting more than 20 hours [19].

Our primary outcome was cesarean birth. With our available sample sizes we had 83% power to find a relative difference of 30% from a baseline cesarean section rate of 26.9%, the overall rate for the randomized controlled trial, with a type I error of 0.05, two sided. Secondary obstetrical outcomes included indication for cesarean birth, length of time from admission to birth, length of first and second stages of labour, use of continuous electronic fetal monitoring, administration of intravenous fluids, augmentation of labour, use of analgesia (nitrous oxide), narcotics, or epidural analgesia), meconium in the amniotic fluid, and rates of postpartum hemorrhage. Newborn outcomes included Apgar score less than 7 at 1 and 5 minutes, resuscitation at birth and admission to a neonatal care unit.

We compared sociodemographic and labour-related characteristics of our study cohorts using the *t*-test for continuous variables and the Chi-square tests for dichotomous variables. A p-value of less than 0.05 was considered statistically significance. We computed relative risks and 95% confidence intervals. All analyses were undertaken using SPSS version 19. 0 (SPSS Statistics, Inc., Chicago, IL).

We received approval for the current study from the University of British Columbia Clinical Research Ethics Board (H10-01721) on August 30, 2010. All participants provided written informed consent for participation in the original trial.

## Results

The study cohorts were comparable with respect to sociodemographic characteristics (Table 1), stature, and labour-related characteristics on admission to hospital (Table 2), with the single exception of gestational age on admission, which differed by less than three days between the two groups. In a multivariate analysis, inclusion of individual prognostic variables in a logistic regression model did not change odds ratios more than 10%. Accordingly we did not adjust for prognostic factors and present unadjusted relative risks.

**Table 1:** Sociodemographic characteristics by study group

**Characteristic**	**Perceived labour ≥ 24 hours**	**Perceived labour < 24 hours**	**p-value**
	**n = 434**	**n = 813**	
	**n (%)**	**n (%)**	
Age, mean ± s.d	28.5 ± 5.1	28.5 ± 5.1	ns
Single parent	18 (4.2)	29 (3.6)	ns
Education			ns
Some high school	40 (9.3)	79 (9.9)	
High school diploma	60 (14.0)	144 (18.1)	
Some post secondary	42 (9.8)	69 (8.7)	
Trade school/college diploma	106 (24.7)	164 (20.6)	
Some university education	41 (9.5)	73 (9.2)	
University education	141 (32.8)	266 (33.5)	
Missing	4	18	
Family Income			ns
<20,000	76 (19.1)	142 (19.5)	
21,000–39,000	95 (23.9)	171 (23.5)	
40,000-59,000	73 (18.3)	138 (19.0)	
>60,000	154 (38.7)	276 (38.0)	
Missing	36	86	
Employment			ns
Full time	270 (63.4)	521 (65.0)	
Part time	39 (9.2)	78 (9.7)	
Unemployed	117 (27.5)	203 (25.3)	
Missing	8	11	
Partner’s employment			ns
Full time	352 (85.4)	655 (86.1)	
Part time	21 (5.1)	36 (4.7)	
Unemployment	39 (9.5)	70 (9.2)	
Missing	22	52	
Ethnicity			ns
Caucasian	187 (43.2)	349 (42.9)	
East Asian	107 (24.7)	175 (21.5)	
South Asian	103 (28.5)	234 (28.8)	
First Nations	11 (2.5)	8 (1.0)	
African Canadian	6 (1.4)	9 (1.1)	
Other	19 (4.4)	38 (4.7)	
Missing	1	0	
English as second language	117 (27.1)	199 (24.9)	ns

**Table 2:** Obstetric characteristics by study group

**Characteristic**	**Perceived labour ≥24 hours**	**Perceived labour <24 hours**	**p-value**
	**n = 434**	**n = 813**	
	**n (%)**	**n (%)**	**n (%)**
Maternal height, cm, mean ± sd	162.9 ± 7.2	163.2 ± 6.8	ns
Pre-pregnancy weight, kg, mean ± sd	60.2 ± 14.8	59.8 ± 13.0)	ns
Weight gain, kg, mean ± sd	16.5 ± 6.1	15.9 ± 6.1)	ns
Gestational age days, mean ± sd	280.3 ± 6.9	277.7 ± 7.1	<.001
Symphysis fundal height cm, mean ± sd	37.1 ± 2.8	36.9 ± 2.8)	ns
Attended prenatal classes	217 (53.2)	396 (51.1)	ns
Doula	21 (5.1)	46 (5.9	ns
Coping on admission			ns
Not distressed	47 (11.5)	95 (12.1)	
Coping with Support	272 (66.8)	500 (63.9)	
Not coping	88 (21.6)	187 (23.9)	
Status of membranes on admission			ns
Intact	300 (69.1)	517 (63.7)	.
Ruptured	29 (6.7)	76 (9.4)	
Unsure	105 (24.2)	219 (27.0)	
Method of early labour assessment			ns
Home visit	225 (51.8)	402 (49.4)	
Telephone triage	209 (48.2)	411 (50.6)	
Meconium in amniotic fluid in labour	49 (11.3)	66 (8.2)	ns

Women who perceived their labour to have lasted 24 hours or longer prior to admission were at excess risk for cesarean section, relative risk (RR) 1.4, 95% Confidence Intervals (CI) (1.15-1.72) (Table 3). Labour dystocia was more often the primary indication for cesarean section in the prolonged pre-hospitalization labour group and fetal distress less often, but these differences were not statistically significant. Women in the prolonged early labour group had on average significantly longer first stages of labour (916 versus 775.5 minutes, p < .001), but second stages were comparable (125.3 versus 118.1 minutes). As well, the elapsed time from admission to birth was significantly longer in the prolonged early labour group, (714.6 versus 580.7 minutes, p < .001). Women who were experiencing pre-admission labour ≥ 24 hours were admitted to hospital significantly more often at a cervical dilation of 3 cm or less, RR 1.21 (1.07-1.36).

**Table 3:** Maternal outcomes by study group

**Outcome**	**Perceived labour ≥24 hours**	**Perceived labour <24 hours**	**RR (95% CI)**
	**n = 434**	**n = 813**	
	**n (%)**	**n (%)**	
Mode of birth			
Vaginal	189 (43.5)	412 (50.7)	0.86 (0.76-0.98)
Forceps or vacuum	122 (28.1)	237 (29.2)	0.96 (0.80–1.16)
Cesarean birth	123 (28.3)	164 (20.2)	1.40 (1.15-1.72)
Primary indication for cesarean			
Dystocia/CPD	99 (80.5)	122 (74.4)	1.08 (0.95-1.23)
Fetal Distress	24 (19.5)	42 (25.6)	0.76 (0.49–1.19)
Length of time from admission to birth (minutes, mean ± sd)	714.6 ± 467.9	580.7 ± 441.6	p < .001
Length of first stage (minutes, mean ± sd)	916.1 ± 501.0	775.5 ± 417.8	p < .001
Length of second stage (minutes, mean ± sd)	125.3 ± 92.5	118.1 ± 97.8	ns
Cervical dilation on admission ≤ 3 cm	223 (52.0)	347 (43.3)	1.21 (1.07-1.36)
Continuous Electronic Fetal Monitoring	316 (72.8)	533 (65.6)	1.11 (1.03-1.20)
IV fluids	384 (88.5)	629 (77.6)	1.14 (1.08–1.20)
Augmentation of labour	331 (76.3)	65 (57.2)	1.33 (1.23–1.44)
Analgesia			
Narcotic (IM or IV)	206 (47.5)	320 (39.4)	1.21 (1.06–1.37)
Nitrous Oxide	294 (67.7)	568 (69.9)	0.97 (0.90–1.05)
Epidural analgesia	327 (75.3)	495 (60.9)	1.18 (1.09–1.28)
Blood loss			
After vaginal birth (>500 cc)	65 (19.9)	112 17.2)	1.21 (0.92-1.59)
After cesarean birth (>1000 cc)	5 (4.1)	6 (3.7)	1.11 (0.353.56)

Women in the prolonged early labour group had a higher risk of experiencing obstetrical interventions including continuous electronic fetal monitoring, administration of intravenous fluids, and augmentation of labour. They more often required intravenous or intramuscular narcotic analgesia RR 1.21 (1.06–1.37) and epidural analgesia RR 1.18 (1.09–1.28).

Between the two groups, there were no differences in rates of Apgar scores less than 7 at 1 and 5 minutes, need for endotracheal suction or oxygen at birth, or admission to a neonatal nursery. Meconium – stained amniotic fluid at birth was significantly more frequent in the prolonged early labour group RR 1.60 (1.09-2.35) (Table 4).

**Table 4:** Newborn outcomes by study group

**Outcomes**	**Perceived labour ≥24 Hours**	**Perceived labour <24 hours**	**RR (95% CI)**
	**n = 434**	**n = 813**	
	**n (%)**	**n (%)**	
Meconium at birth	58 (13.4)	71 (8.8)	1.60 (1.09–2.35)
Apgar score <7 at 1 min	59 (13.6)	96 (11.8)	1.15 (0.85–1.56)
Apgar score <7 at 5 min	4 (0.9)	6 (0.7)	1.25 (0.35–4.40)
Resuscitation at birth			
Suction with endotracheal tube	35 (8.1)	56 (6.9)	1.17 (0.78–1.76)
Intermittent positive pressure	53 (12.3)	79 (9.8)	1.26 (0.91–1.75)
Admit to observation or intensive care nursery	33 (7.6)	54 (6.6)	1.14 (0.75–1.74)

The positive predictive value for perceived labour prior to hospital admission ≥ 24 hours in predicting cesarean section was 28.3%. This indicator has low sensitivity, meaning that its application would fail to pick up a substantial number of women who were destined to have a cesarean section. The negative predictive value was 79.8%. This indicates that a negative finding (labour < 24 hours prior to admission) is accurate 80% of the time in identifying women who would not have a cesarean delivery.

## Discussion

This study demonstrates, for the first time, that women’s perceptions that their labour has lasted 24 hours or longer at the time of admission to hospital increases the risk of admission in the latent phase of labour, cesarean delivery, and exposure to other obstetrical interventions.

Early admission to hospital is a well-described risk factor for obstetrical interventions and birth by cesarean [7,8,13], however there is limited literature on the sequelae of prolonged latent phase labour. The study of latent phase labour is hampered by a lack of consensus on the definition of its onset, making the diagnosis of prolonged labour problematic.

Maghoma and Buchmann studied early labour among 250 healthy women [20]. They enrolled both multiparous and nulliparous women and defined prolonged early labour as greater than 8 hours, measured from the time of the first clinical assessment in labour to the clinical assessment of active phase. Consistent with our findings, cesarean birth was significantly more frequent in the prolonged early labour group as was augmentation of labour. A similar study by Chelmow et al. defining prolonged latent phase as >12 hours for nulliparous women and > 6 hours for multiparous women reported that prolonged latent phase was independently associated with an increased incidence of abnormal progress in the active phase, and cesarean delivery [21].

Maghoma and Buchmann found a similar increase in risk of meconium as well as a significant difference in 5 minute Apgars less than 7, and admission to a level II (observation) or level III (intensive care) nursery for women with prolonged early labour [20]. Chelmow et al. reported a higher frequency of low Apgar scores, and need for newborn resuscitation in the group with prolonged latent phase [21].

The increase in interventions observed in the women with prolonged pre-hospital labour may be due to dysfunctional labour manifesting in the latent phase or may be iatrogenic, subsequent to longer time spent in hospital. The observational nature of our study does not allow for causal inferences to be drawn from our data. We did observe, however, that among women who stated their labour had started ≥ 24 hours prior to admission, the cesarean rate for those whose labour was augmented was significantly lower than among those who were not augmented (31.7% versus 14.5%, p = .005), supporting the notion that recognition and treatment of prolonged latent phase labour may be of value in preventing cesarean section. A randomized trial of active management of labour among women with prolonged latent phase labour, including oxytocin augmentation, with standardized protocols for other aspects of labour management may answer this question.

## Conclusion

In this retrospective cohort study, women’s perceptions of labour lasting 24 hours or more at the time of presentation to hospital for birth was associated with a 40% increase in risk for giving birth by cesarean. These findings are generalizable to healthy nulliparous women experiencing uncomplicated pregnancies. Women experiencing prolonged pre-hospital early labour were also at a higher risk for most obstetrical interventions and postpartum hemorrhage after a vaginal birth.

Asking women about the duration of their labour, without practitioner-defined parameters, may serve as a useful screening device for women who may potentially benefit from close observation and more active management of labour. A high negative predictive value means that a negative response to the question about length of pre-hospital labour lasting 24 hours or longer could be used to identify women at minimal risk for cesarean delivery, for whom efforts to promote vaginal delivery should be intensified. In addition, this question is simple to ask and avoids the controversies surrounding clinician-defined onset of labour.

## Competing interests

The authors declare that they have no competing interests.

## Authors’ contributions

PJ conceived the design of the study, participated in data analysis and writing the manuscript. SW was the primary data analyst and wrote the initial draft of the manuscript. Both authors read and approved the final manuscript.

## Pre-publication history

The pre-publication history for this paper can be accessed here:

http://www.biomedcentral.com/1471-2393/14/182/prepub

## References

[B1] Canadian Institute for Health Information Health Indicators 2012

[B2] Hamilton B Martin J Ventura S Births, Preliminary Data for 2010 Natl Vital Stat Rep 2011 60 2 1 24DHHS Publication No. (PHS) 2012–1120 24979973

[B3] Mamun A Calloway L O’Callaghan M Williams G Najman J Alati R Clavarino A Lawlor D Associations of maternal pre-pregnancy obesity and excess pregnancy weight gains with adverse pregnancy outcomes and length of hospital stay BMC Preg Childbirth 2001 11 doi:10.1186/1471-2393-1111-1162 10.1186/1471-2393-11-62 PMC3178538 21892967

[B4] Joseph K Young D Dodds L O'Connell C Allen V Chandra S Allen A Changes in maternal characteristics and obstetric practice and recent increases in primary cesarean delivery Obstet Gynecol 2003 102 791 800 10.1016/S0029-7844(03)00620-3 14551010

[B5] Henderson J Mugford M Chamberlain G, Wraight A, Crowley P An Economic Evaluation Of Homebirths Home Births: the Report of the 1994 Confidential Enquiry for the National Birthday Trust Fund 1997 London, UK: Parthenon 191 211

[B6] Henderson J Petrou S Economic implications of home births and birth centers: a structured review Birth Issues Perinat Care 2008 35 2 136 145 10.1111/j.1523-536X.2008.00227.x 18507585

[B7] Bailit J Dierker L Blanchard M Mercer B Outcomes of women presenting in active versus latent phase of spontaneous labour Obstet Gynecol 2005 2005 77 79 15625145 10.1097/01.AOG.0000147843.12196.00

[B8] Wuitchik M Bakal D Lipshitz J The clinical significance of pain and cognitive activity in latent labor Obstet Gynecol 1989 73 1 35 42 2909041

[B9] Holmes P Oppenheimer L Wen S The relationship between cervical dilatation at initial presentation in labour and subsequent intervention Br J Obstet Gynaecol 2001 108 1120 1124 10.1111/j.1471-0528.2003.00265.x 11762649

[B10] Hemminki E Simukka R The timing of hospital admission and progress of labour Eur J Obstet Gynecol Reprod Biol 1986 22 85 94 10.1016/0028-2243(86)90093-6 3721051

[B11] Klein M Kelly A Kaczorowski J Gryzbowski S The effect of family physician timing of maternal admission on procedures in labour and maternal and infant morbidity J Obstet Gynaecol Can 2004 26 7 641 645 15248933 10.1016/s1701-2163(16)30611-9

[B12] Jackson D Lang J Ecker J Swartz W Heeren T Impact of collaborative management and early admission in labor on method of delivery J Obstet Gynecol Neonatal Nurs 2003 32 2 147 157 10.1177/0884217503252045 12685666

[B13] Indraccolo U Filippo D Iorio D Marinoni E Roselli D Indraccolo S Effect of epidural analgesia on operative vaginal birth rate Clin Exp Obstet Gynecol 2011 38 3 221 224 21995150

[B14] Janssen P Still K Klein M Singer J Carty E Liston R Zupancic J Early labour assessment and support at home vs. telephone triage Obstet Gynecol 2006 108 1463 1469 10.1097/01.AOG.0000247644.64154.bb 17138781

[B15] Roman H Carayol M Watier L Ray C Breart G Goffinet F Planned vaginal delivery of fetuses in breech presentation at term: prenatal determinants predictive of elevated risk of cesarean dleivery during labor Eur J Obstet Gynecol Reprod Biol 2008 138 1 14 22 10.1016/j.ejogrb.2007.06.019 17689853

[B16] Seaman S Bartlett J White I Multiple imputation of missing covariates with non-linear effects and interactions: an evaluation of statistical methods BMC Med Res Methodol 2012 12 1 doi:10.1186/1471-2288-1112-1146 10.1186/1471-2288-12-46 PMC3403931 22489953

[B17] Hodnett E Osborn R Hannah M Willan A Stevens B Weston J Ohlsson A Gafni A Muir H Myhr T Stremier R Nursing Supportive Care in Labor Trial Group Effects of nurses as providers of birth labor support in North American Hospitals J Am Med Assoc 2002 288 11 1373 1381 10.1001/jama.288.11.1373 12234231

[B18] Torloni M Betran A Souza J Widmer M Allen T Gulmezoglu M Merialdi M Classifications for cesarean section:a systematic review PLoS One 2011 6 1 e14566 10.1371/journal.pone.0014566 21283801 PMC3024323

[B19] The American College of Obstetricians and Gynecologists Obstetric Care Consensus: Safe prevention of the primary cesarean delivery Obstet Gynecol 2014 123 3 693 710 10.1097/01.AOG.0000444441.04111.1d 24553167

[B20] Maghoma J Buchmann E Maternal and fetal risks associated with prolonged latent phase of labour J Obstet Gynaecol 2002 22 1 16 18 10.1080/01443610120101637 12521720

[B21] Oumeish O The philosophical, cultural, and historical aspects of complementary, alternative, and unconventional, and integrative medicine in the Old World Arch Dermatol 1998 134 1373 1386 9828871 10.1001/archderm.134.11.1373

